# Misoprostol Induced Expulsion of Fetus Following Diagnosis of Anencephaly with Cardiac Abnormalities on Ultrasound: A Case Report

**DOI:** 10.31729/jnma.6402

**Published:** 2021-04-30

**Authors:** Hari Kishor Shrestha, Suphatra Koirala, Ingima Shrestha

**Affiliations:** 1 Department of Radiology, Om hospital and Research Center, Chabahil, Kathmandu, Nepal; 2 Department of Obstetrics and Gynaecology, Om hospital and Research Center, Chabahil, Kathmandu, Nepal; 3 Om hospital and Research Center, Chabahil, Kathmandu, Nepal

**Keywords:** *anencephaly*, *cardiac anomalies*, *ultrasound*

## Abstract

Anencephaly is a condition in which there is an absence of skull and brain tissues. Absence of cranial vault mainly results because of defective neurulation. Absence of cerebral tissues may cause diminished heart size, due to decreased heart load causing various cardiac abnormalities. Here, we report a case of a primigravida lady at 17 weeks of gestation with misoprostol induced expulsion of fetus after ultrasonography revealed absence of brain tissue and calvarium above the orbits suggesting anencephaly. A 300g fetus was delivered which confirmed the ultrasound findings. The patient was discharged with advice for intake of folic acid beginning from 3 months before conception in future pregnancies. Neural tube defects can manifest within approximately 28 days of gestation which highlights the importance of oral folic acid intake before pregnancy.

## INTRODUCTION

Anencephaly is a neural tube defect where the proximal tube fails to close. There is an open defect at the calvarium. Anencephaly occurs in 1:1,000 to 1:20,000 infants.^1^ Most of the anencephaly shows a multifactorial pattern of inheritance, with interaction of multiple genes as well as environmental factors.^2^ Women who are planning to conceive should be informed about the importance of folic acid in fetal development and advised to take 400mcg/day of folic acid supplements.^3^

## CASE REPORT

A 22-year-old primigravida lady from Kathmandu at 17 weeks and 3 days of gestation presented to the outpatient department of our institution for her regular antenatal checkup (ANC) visit. The first day of her last menstrual period (LMP) was on Feb 27^th^ 2020, and her expected date of delivery was 3^rd^ December 2020.

**Figure 1.**
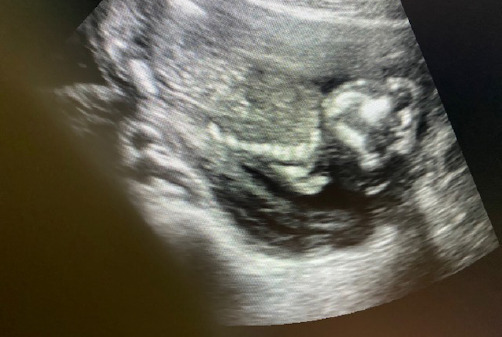
Ultrasound images showing the absent calvaria and exposed brain tissue.

During her routine antenatal ultrasound scan (USG), a singleton live intrauterine fetus at 17 weeks with absent brain tissue above the orbits (toad sign) was seen (Figure 1).

Normal fetal movements and a regular cardiac activity were noted. The fetal femoral length was 27mm and the abdominal circumference was 107mm. The fetus's thorax, spine, nose, eyes, abdominal wall and stomach were normal. Four chamber view was seen in the heart. A three vessel cord was seen. Urinary bladder, upper and lower limbs were also seen.

Posterior placentation with first grade maturity was noted without calcifications or infarcts. Amniotic fluid was normal in amount and the cervical canal was 36 mm without dilation. Based on the USG findings, the patient was advised for termination of pregnancy and was admitted on the same day at 1:00 pm. Following admission, the patient's vitals were being monitored every hourly.

The patient was afebrile and stable. Abdomen was soft and non-tender, and she was watched for expulsion. At 4:00 pm, she complained of 3 episodes of diarrhoea but she was otherwise normal.

A per vaginal exam done at 6:30pm revealed that the membranes had been ruptured and the os was 1cm dilated. At 7:02pm, she was prescribed Tablet Misoprostol 400mcg via buccal route at to be given at 8 pm along with an intravenous infusion of oxytocin 5 IU in 500ml of dextrose at 40 drops per minute. Another per vaginal examination done at 7:13pm found a 2cm dilated os with ruptured membranes. The rate of infusion of oxytocin was reduced to 10 drops per minute.

**Figure 2.**
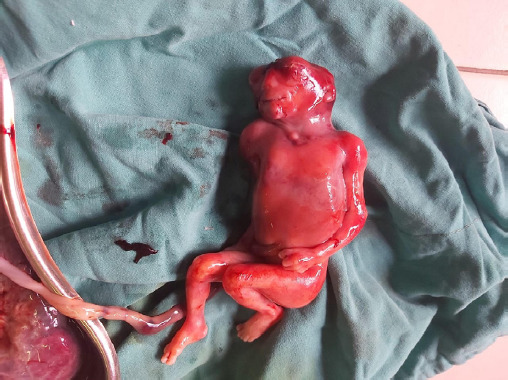
Fetus with anencephaly and low set ears.

At 9pm, the patient was taken to operation theatre for expulsion of fetus. She was put on lithotomy position, and the bladder was evacuated. She was encouraged to push in each contraction. An anencephalic fetus weighing about 300 gm was delivered at 9:00pm and the placenta was delivered by controlled cord traction (Figure 2).

Hemostasis was secured and a vaginal toileting was done. Tablet misoprostol 600mcg was inserted per rectum immediately. The total estimated blood loss was about 50ml.

At 10:56pm, the patient was shifted to the ward. Her post-delivery period was uneventful. The patient was discharged 2 days after delivery.

## DISCUSSION

We discussed a case of a fetus delivered at 17 weeks and 3 days of gestation after misoprostol induction for USG findings of anencephaly. Although fetal cardiac activity and fetal movements were noted in the USG, fetuses with such severe neural tube defects are not compatible with life. Even if they are born, they may survive for a few hours to days.

The central nervous system develops from the neural plate at the beginning of the third week of gestation. The lateral margins of the plate thicken, elevate and join in the midline to close in cranial to caudal direction. The cranial neuropore closes at the 25^th^ day and caudal at about the 27^th^ day of gestation. The failure of this closure of the neural tube gives rise to neural tube defects.

Anencephaly is considered as a lethal diagnosis as it has been reported to be 100% lethal in the first year of life.^4,5^ Congenital lack of mesenchymal migration in the fourth week of gestation leading to absence of the calvarium and abnormal development of the cortical structures results in the development of anencephaly.^6^ The early recognition of this diagnosis allowed pregnancy decisions to be made earlier. Ultrasound is the ideal imaging method for the early detection of fetal anomalies given its high diagnostic capacity, non-invasiveness, rapid detection, low cost, and availability. Moreover, ultrasound offers the advantage of earlier detection beginning at 10 weeks gestation to allow more comprehensive parent counseling and earlier decisions regarding the future of the pregnancy.^7,8^

Chandrupatla et al. reported a similar case of anencephaly diagnosed at USG and confirmed after delivery of fetus.^9^
